# Associations of the *APOC3* rs5128 polymorphism with plasma APOC3 and lipid levels: a meta-analysis

**DOI:** 10.1186/s12944-015-0027-0

**Published:** 2015-04-18

**Authors:** Yongyan Song, Liren Zhu, Mudwari Richa, Ping Li, Yang Yang, Suping Li

**Affiliations:** Department of Medical Biochemistry, School of Preclinical Medicine, North Sichuan Medical College, Nanchong, 637000 P R. China; School of Clinical Medicine, North Sichuan Medical College, Nanchong, 637000 P R. China; Department of Nuclear Medicine, Affiliated Hospital of North Sichuan Medical College, Nanchong, 637000 P R. China

**Keywords:** Meta-analysis, *APOC3*, rs5128, Polymorphism, Lipid

## Abstract

**Background:**

Studies of the association between the apolipoprotein C3 gene (*APOC3*) rs5128 polymorphism and plasma levels of apolipoprotein C3 (APOC3) and lipids have reported apparently conflicting findings. This meta-analysis aimed to investigate the associations of the rs5128 polymorphism with fasting APOC3 and lipid levels.

**Methods:**

The following information was abstracted for each study: ethnicity, age, sex, health condition, sample size, genotyping and lipid assay methods, mean and standard deviation or standard error by genotypes for APOC3 and lipid variables. There were 42 eligible studies with 23846 subjects included in this meta-analysis. A dominant model was used for this meta-analysis.

**Results:**

The results showed that the carriers of the variant allele G had higher levels of APOC3 [standardized mean difference (SMD): 0.22, 95% confidence interval (CI): 0.12-0.31, *P* < 0.00001], triglycerides (TG) (SMD: 0.33, 95% CI: 0.23-0.44, *P* < 0.00001), total cholesterol (TC) (SMD: 0.15, 95% CI: 0.09-0.22, *P* < 0.00001), and low-density lipoprotein cholesterol (LDL-C) (SMD: 0.11, 95% CI: 0.04-0.17, *P* = 0.001) than the non-carriers. No significant association between the *APOC3* rs5128 polymorphism and lower levels of high-density lipoprotein cholesterol (HDL-C) was detected under the dominant model (SMD: −0.03, 95% CI: −0.06-0.01, *P* = 0.156).

**Conclusions:**

The results from the present meta-analysis demonstrate a significant association between the *APOC3* rs5128 polymorphism and higher levels of APOC3, TG, TC and LDL-C, but further studies are needed to elucidate the underlying mechanisms.

**Electronic supplementary material:**

The online version of this article (doi:10.1186/s12944-015-0027-0) contains supplementary material, which is available to authorized users.

## Introduction

Cardiovascular disease (CVD) is now the leading cause of death worldwide, both in developed countries and in many developing countries [1]. After decades of study, a number of CVD risk factors have been identified. Among these risk factors, dyslipidemia, characterized by elevations of triglycerides (TG), total cholesterol (TC) and/or low-density lipoprotein cholesterol (LDL-C), or reduction of high-density lipoprotein cholesterol (HDL-C) levels, accounts for at least 50% of the population-attributable risk for CVD [2]. In recent years, much has been learned about the chromosomal regions and specific genes that influence plasma lipid or lipoprotein levels [3]. However, due to various reasons, it is difficult to identify the susceptibility genes and most associations have not been replicated across studies.

The apolipoprotein C3 gene (*APOC3*) is a member of the *APOA1/C3/A4/A5* gene cluster and located on chromosome 11q23, an area in strong linkage with lipid metabolism [3]. Apolipoprotein C3 (APOC3) is an essential constituent of TG-rich particles including chylomicron and very low-density lipoprotein (VLDL), to a less extent of high-density lipoprotein (HDL). Evidence from gene engineering studies suggested that the main role of APOC3 could be involved in the regulation of plasma TG levels [4,5]. Additional copies of human *APOC3* in transgenic pig were associated with hypertriglyceridemia (HTG) [4], whereas APOC3 deficiency prevents hyperlipidemia induced by apolipoprotein E gene overexpression [5]. *In vitro*, APOC3 was found to delay the catabolism of VLDL by inhibiting the lipoprotein lipase (LPL) which is the rate-limiting enzyme for TG hydrolysis [6]. Furthermore, it also interferes the apolipoprotein E-mediated remnant removal by displacement of apolipoprotein E from VLDL particles *in vivo* [7,8]. APOC3 also play important roles in modulating other lipid variables [9]. Given its role in plasma lipid metabolism, *APOC3* is considered a candidate gene for dyslipidemia.

Of several variants within *APOC3*, a transversion from C to G in the 3' untranslated region (3'UTR) of exon 4 results in a rs5128 polymorphism (also known as SstI, sacI, 3238C > G or 3175C > G). A recent meta-analysis [10] demonstrated that the rs5128 polymorphism is associated with CVD, but whether this polymorphism is also associated with dyslipidemia remains to be further examined. A large body of studies has investigated the associations of this polymorphism with plasma APOC3 and lipid levels [11-52]. Some studies found that this polymorphism is associated with higher plasma levels of APOC3 [14,20,27,41,42], TG [18,20,23,25,28,30,32,33,38,41,42,44,46,51], TC [18,32,51] and LDL-C [30,51], and lower levels of HDL-C [24]. However, the results from other studies were conflicting and inconclusive. In this paper, a meta-analysis was performed on previous reports to investigate the associations of the rs5128 polymorphism with APOC3 and fasting lipid levels. Our analysis results can provide the opportunity to elucidate the interrelationship among the rs5128 polymorphism, dyslipidemia and CVD.

## Methods

### Identification and eligibility of relevant studies

All articles published before May 2014 on the associations of the *APOC3* rs5128 polymorphism with plasma APOC3 and lipid levels were identified. The language was limited to English. A comprehensive search of the literature was carried out by using PubMed and EMBASE. The keywords used for this search were “APOC3 OR APOC-III OR apolipoprotein C-III OR apolipoprotein CIII OR apolipoprotein C3 OR APOC3 OR APO C3” concatenated with “polymorphism OR variant OR SNP OR mutation”. This meta-analysis is limited to APOC3 and the plasma lipid variables including TG, TC, LDL-C, and HDL-C. The studies that fulfilled the following criteria were included: (1) data reported on fasting variables; (2) data reported on APOC3 and/or at least one of the four plasma lipid variables; (3) pre-intervention baseline data were used in interventional studies; (4) the studies in which mean plasma lipid levels and standard deviations (SD) or standard errors (SE) by genotype were available. In order to find other published work which was not indexed by PubMed and EMBASE, all references cited in the included articles were reviewed. Case reports, review articles, abstracts, animal studies, reports with incomplete data, and studies based on pedigree data were excluded.

### Data extraction

The irrelevant and overlapping studies were excluded after being reviewed independently by two reviewers using a structured data collection form. The results were compared, and the disagreements were resolved by discussion. Regarding the overlapping articles, only those publications that reported the most extensive information were included. For each study included in the present meta-analysis, the following information was extracted: first author, year of publication, ethnicity, country of origin, age, sex, health condition, genotyping and lipid assay methods, sample size, mean APOC3 or lipid variables and SD or SE by genotypes.

### Statistical analysis

All data were presented as mean ± SD in this analysis. For those included articles in which mean ± SE was given, the value of the SD was calculated. The STATA software package v 10.0 (Stata Corporation, College Station, TX) was used for the meta-analysis. Due to the low frequencies of the GG genotype, a dominant model [(CG + GG) versus CC] was employed to ensure adequate statistical power. When data was presented for more than one subpopulation (for example, female or male subjects, the subjects with CVD or type 2 diabetes, the subjects from different ethnicity) in one article, each subpopulation was treated as a separate comparison in this meta-analysis. In addition, subgroup analyses were conducted by age, gender, ethnicity and health condition. Age subgroups were defined as adults and children (under 18 years old). Ethnic subgroups were defined as Caucasian, Asian, and the populations of other ethnic origins. The meta-analyses on the subgroup were only performed with at least four comparisons to ensure adequate statistical power.

The pooled standardized mean difference (SMD) and its 95% confidence interval (CI) were used to assess the differences of the variables between the genotypes. A random effects model was used for all analyses because both between-study and within-study heterogeneity is considered in this model; it provides a more conservative evaluation of the significance of the association than the fixed effects model [53]. Heterogeneity between studies was tested by Cochran's χ^2^-based Q-statistic at a significance level of *P* < 0.05. Galbraith plot was used to detect the potential sources of heterogeneity, and the pooled SMD was recalculated after removal of the outlier studies identified in the plot. The populations in the studies were tested for Hardy-Weinberg equilibrium (HWE) by χ^2^ test; the significance level is defined as α < 0.05. Publication bias was assayed by Egger's linear regression test [54], and a significance level of 0.05 was used to indicate the presence of potential publication bias.

## Results

### Characteristics of the included studies

Initial search of the literature yielded 646 publications. Five hundred and eighteen studies were excluded according to title and abstract. Then full text articles were retrieved and assessed on the basis of the inclusion criteria. Eighty-six papers were ineligible for the following reasons: 57 papers did not provide complete data for this meta-analysis, 24 papers presented data on other polymorphisms, 2 papers had subjects overlap with other publications, and 3 studies were based on pedigree data. In the end, 42 studies were selected for this meta-analysis.

The characteristics of the 42 included studies were summarized in Table 1. Of these, 14 studies [11,13,14,19,20,22-24,26,27,30,33,41,42], 40 studies [11-13,15-39,41-52], 31 studies [11,13,15,17-19,21-24,26,28-36,38-40,44,45,47-52], 27 studies [17,18,21-24,26,28,30-34,36-41,44,45,47-52] and 34 studies [11,15-19,21-24,26,28,30-41,43-52] presented the data on APOC3, TG, TC, LDL-C and HDL-C, respectively. Twenty-six studies [12,14-18,20-23,25-30,32,35,39-41,43,44,46-48], 10 studies [11,19,24,33,34,37,38,42,45,49] and 6 studies [13,31,36,50-52] involved Caucasians, Asians, and the subjects of other ethnic origins, respectively. Seven studies [12,16,21,22,30,35,50] only involved males, and the other 35 studies involved both males and females, among which 9 studies [14,23,26,28,29,36,37,43,47] separately provided data for males and females. Three studies [20,36,47] involved children. Four studies [18,33,38,48] and 9 studies [12,15-17,19,34,35,38,50] involved type 2 diabetes and CVD, respectively. Twenty-three studies [12,14-17,19,23,25,26,28,29,34-38,43,45,47-50,52] separately provided data for more than one subpopulation, and each subpopulation was treated as a separate comparison. Genotype distribution in 6 populations or subpopulations [25,27,31,38,50,51] significantly deviated -from HWE. The units of APOC3 or plasma lipids used in the eligible studies included mg/dL or mmol/L. The complete plasma APOC3 and lipid data by genotype can be found in Additional file 1: Table S1.

**Table 1:** **Characteristics of individual studies included in the meta-analysis**

**First author, reference**	**Year**	**Ethnicity**	**Gender**	**Study population**	**Outcomes**
Aburatani [11]	1988	Asian	M/F	Subjects with hyperlipidemia and controls	APOC3, TG, TC, HDL-C
Paulweber1 [12]	1988	Caucasian	M	Patients with CVD	TG
Paulweber2 [12]	1988	Caucasian	M	Subjects without CVD	TG
Ahn [13]	1991	Other	M/F	Random subjects	APOC3, TG, TC
Shoulders1 [14]	1991	Caucasian	M	Healthy subjects	APOC3
Shoulders2 [14]	1991	Caucasian	F	Healthy subjects	APOC3
Ordovas1 [15]	1991	Caucasian	M/F	Patients with CVD	TG, TC, HDL-C
Ordovas2 [15]	1991	Caucasian	M/F	Healthy population	TG, TC, HDL-C
Tybjaerg-Hansen1 [16]	1993	Caucasian	M	Subjects with CVD	TG, HDL-C
Tybjaerg-Hansen2 [16]	1993	Caucasian	M	Subjects without CVD	TG, HDL-C
Miettinen1 [17]	1994	Caucasian	M/F	Subjects with CVD	TG, TC, LDL-C, HDL-C
Miettinen2 [17]	1994	Caucasian	M/F	Subjects without CVD	TG, TC, LDL-C, HDL-C
Rigoli [18]	1995	Caucasian	M/F	Patients with type 2 diabetes	TG, TC, LDL-C, HDL-C
Bai1 [19]	1995	Asian	M/F	Subjects with CVD	APOC3, TG, TC, HDL-C
Bai2 [19]	1995	Asian	M/F	Subjects without CVD	APOC3, TG, TC, HDL-C
Shoulders [20]	1996	Caucasian	M/F	Random healthy children	APOC3, TG
López-Miranda [21]	1997	Caucasian	M	Random healthy youth	TG, TC, LDL-C, HDL-C
Kee [22]	1999	Caucasian	M	Random subjects	APOC3, TG, TC, LDL-C, HDL-C
Dallongeville1 [23]	2000	Caucasian	M	Random subjects	APOC3, TG, TC, LDL-C, HDL-C
Dallongeville2 [23]	2000	Caucasian	F	Random subjects	APOC3, TG, TC, LDL-C, HDL-C
Wu [24]	2000	Asian	M/F	Random subjects without CVD	APOC3, TG, TC, LDL-C, HDL-C
Waterworth1 [25]	2000	Caucasian	M/F	Non-smokers	TG
Waterworth2 [25]	2000	Caucasian	M/F	Exsmokers (cessation for minimum of 1 year)	TG
Waterworth3 [25]	2000	Caucasian	M/F	Current smokers	TG
Russo1 [26]	2001	Caucasian	M	Framingham Offspring Study	APOC3, TG, TC, LDL-C, HDL-C
Russo2 [26]	2001	Caucasian	F	Framingham Offspring Study	APOC3, TG, TC, LDL-C, HDL-C
Olivieri [27]	2002	Caucasian	M/F	Subjects with or without CVD	APOC3, TG
Corella1 [28]	2002	Caucasian	M	Random healthy subjects	TG, TC, LDL-C, HDL-C
Corella2 [28]	2002	Caucasian	F	Random healthy subjects	TG, TC, LDL-C, HDL-C
Rodrigo1 [29]	2002	Caucasian	M	Patients undergoing kidney transplantation	TG, TC
Rodrigo2 [29]	2002	Caucasian	F	Patients undergoing kidney transplantation	TG, TC
Couillard [30]	2003	Caucasian	M	Abdominally obese subjects	APOC3, TG, TC, LDL-C, HDL-C
Brown [31]	2003	Other	M/F	Random subjects	TG, TC, LDL-C, HDL-C
Espino-Montoro [32]	2003	Caucasian	M/F	Hypertensive patients	TG, TC, LDL-C, HDL-C
Chen [33]	2004	Asian	M/F	Patients with type 2 diabetes	APOC3, TG, TC, LDL-C, HDL-C
Chhabra1 [34]	2004	Asian	M/F	Patients with CVD	TG, TC, LDL-C, HDL-C
Chhabra2 [34]	2004	Asian	M/F	Subjects without CVD	TG, TC, LDL-C, HDL-C
Liu1 [35]	2004	Caucasian	M	Subjects with CVD	TG, TC, HDL-C
Liu2 [35]	2004	Caucasian	M	Subjects without CVD	TG, TC, HDL-C
de França1 [36]	2005	Other	M	Healthy children	TG, TC, LDL-C, HDL-C
de França2 [36]	2005	Other	F	Healthy children	TG, TC, LDL-C, HDL-C
Arai1 [37]	2005	Asian	M	Random subjects	TG, LDL-C, HDL-C
Arai2 [37]	2005	Asian	F	Random subjects	TG, LDL-C, HDL-C
Liu1 [38]	2005	Asian	M/F	Patients with CVD	TG, TC, LDL-C, HDL-C
Liu2 [38]	2005	Asian	M/F	Patients with type 2 diabetes	TG, TC, LDL-C, HDL-C
Liu3 [38]	2005	Asian	M/F	Subjects without CVD and type 2 diabetes	TG, TC, LDL-C, HDL-C
Islam [39]	2005	Caucasian	M/F	Random subjects	TG, TC, LDL-C, HDL-C
Stancáková [40]	2006	Caucasian	M/F	Patients with dyslipidemia of metabolic syndrome	TC, LDL-C, HDL-C
Herron [41]	2006	Caucasian	M/F	Random subjects	APOC3, TG, LDL-C, HDL-C
Huang [42]	2006	Asian	M/F	Subjects with hypertriglyceridemia or normal	APOC3, TG
Fiegenbaum1 [43]	2007	Caucasian	M	Random healthy subjects	TG, HDL-C
Fiegenbaum2 [43]	2007	Caucasian	F	Random healthy subjects	TG, HDL-C
Nieminen [44]	2007	Caucasian	M/F	Random youth	TG, TC, LDL-C, HDL-C
Parzianello1 [45]	2008	Asian	M/F	Subjects with hypertriglyceridemia	TG, TC, LDL-C, HDL-C
Parzianello2 [45]	2008	Asian	M/F	Subjects free of hypertriglyceridemia	TG, TC, LDL-C, HDL-C
Dallongeville [46]	2008	Caucasian	M/F	Random subjects	TG, HDL-C
Ruiz1 [47]	2008	Caucasian	M	Random healthy children	TG, TC, LDL-C, HDL-C
Ruiz2 [47]	2008	Caucasian	F	Random healthy children	TG, TC, LDL-C, HDL-C
Smith1 [48]	2009	Caucasian	M/F	Patients with type 2 diabetes	TG, TC, LDL-C, HDL-C
Smith2 [48]	2009	Caucasian	M/F	Subjects free of type 2 diabetes	TG, TC, LDL-C, HDL-C
Yiyang1 [49]	2010	Asian	M/F	Random subjects	TG, TC, LDL-C, HDL-C
Yiyang2 [49]	2010	Asian	M/F	Random subjects	TG, TC, LDL-C, HDL-C
Sediri1 [50]	2011	Other	M	Males with CVD	TG, TC, LDL-C, HDL-C
Sediri2 [50]	2011	Other	M	Males without CVD	TG, TC, LDL-C, HDL-C
Abd El-Aziz3 [51]	2011	Other	M/F	Patients with CVD and controls	TG, TC, LDL-C, HDL-C
Bandegi1 [52]	2011	Other	M/F	Subjects with primary hyperlipidemia	TG, TC, LDL-C, HDL-C
Bandegi2 [52]	2011	Other	M/F	Normolipidemic subjects	TG, TC, LDL-C, HDL-C

CVD: cardiovascular disease, M: male, F: female, TG: triglyceride, TC: total cholesterol, LDL-C: low-density lipoprotein cholesterol, HDL-C: high-density lipoprotein cholesterol.

### Summary statistics

Sixty-seven comparisons were distinguished according to the categories such as age, gender, smoking status, and health condition. Of these, 18, 64, 49, 42 and 54 comparisons were included for comparing the differences in APOC3, TG, TC, LDL-C and HDL-C, respectively (Table 2). Totally, 23846 subjects were enrolled in this meta-analysis, 74% of them (17592 subjects) have the CC genotype, and 26% of them (6254 subjects) have the GC or GG genotype. 6523, 23528, 15891, 15310 and 19527 subjects were included in comparing the differences in APOC3, TG, TC, LDL-C and HDL-C, respectively (Additional file 1: Table S1).

**Table 2:** **Meta-analysis of the** ***APOC3*** **rs5128 polymorphism and plasma APOC3 and lipids association**

**Groups or subgroups**	**Comparisons (n)**	**Q test** ***P*** **value**	**SMD (95% CI)**	***P***
APOC3				
All	18	0.004	0.22(0.12, 0.31)	<0.00001
All in HWE	15	0.007	0.20(0.09, 0.32)	0.001
Healthy	12	0.001	0.22(0.08, 0.35)	0.001
Male	5	0.135	0.11(−0.05, 0.27)	0.170
Caucasian	11	<0.00001	0.26(0.11, 0.40)	0.001
Asian	6	0.981	0.15(0.02, 0.29)	0.029
TG				
All	64	<0.00001	0.33(0.23, 0.44)	<0.00001
All in HWE	48	<0.00001	0.31(0.20, 0.42)	<0.00001
Male	19	0.411	0.14(0.07, 0.21)	<0.00001
Female	8	0.062	0.17(0.04, 0.29)	0.011
Children	5	<0.00001	0.53(−0.37, 1.44)	0.249
Caucasian	38	<0.00001	0.39(0.24, 0.54)	<0.00001
Asian	17	0.302	0.18(0.12, 0.25)	< 0.00001
Other	9	< 0.00001	0.37(0.04, 0.69)	0.026
Healthy	39	< 0.00001	0.24(0.13, 0.35)	< 0.00001
CVD	9	0.188	0.29(0.16, 0.43)	< 0.00001
TC				
All	49	< 0.00001	0.15(0.09, 0.22)	< 0.00001
All in HWE	41	0.06	0.12(0.07, 0.18)	< 0.00001
Male	13	0.52	0.06(−0.01, 0.14)	0.105
Female	6	0.23	0.20(0.07, 0.33)	0.002
Children	4	0.062	0.30(0.05, 0.55)	0.021
Caucasian	26	0.08	0.11(0.05, 0.18)	0.001
Asian	14	0.19	0.15(0.06, 0.24)	0.001
Other	9	< 0.00001	0.25(−0.03, 0.53)	0.081
Healthy	39	0.53	0.10(0.06, 0.14)	< 0.00001
CVD	7	0.46	0.15(0.04, 0.26)	0.008
Type 2 diabetes	4	0.18	0.24(0.05, 0.43)	0.013
LDL-C				
All	42	< 0.00001	0.11(0.04, 0.17)	0.001
All in HWE	34	0.043	0.07(0.01, 0.13)	0.021
Male	11	0.069	0.06(−0.10, 0.12)	0.847
Female	6	0.197	0.13(0.02, 0.24)	0.021
Children	4	0.109	0.28(0.05, 0.51)	0.016
Caucasian	21	0.134	0.06(0.00, 0.13)	0.051
Asian	13	0.262	0.09(0.01, 0.16)	0.021
Other	8	< 0.00001	0.22(−0.06, 0.50)	0.119
Healthy	28	0.159	0.06(0.01, 0.11)	0.012
CVD	4	0.115	0.07(−0.13, 0.27)	0.482
Type 2 diabetes	4	0.996	0.22(0.08, 0.36)	0.002
HDL-C				
All	54	0.314	−0.03(−0.06, 0.01)	0.156
All in HWE	42	0.195	−0.01(−0.06, 0.03)	0.597
Male	16	0.474	−0.01(−0.13, 0.00)	0.062
Female	7	0.663	−0.03(−0.11, 0.06)	0.540
Children	4	0.989	0.03(−0.13, 0.19)	0.733
Caucasian	30	0.763	−0.06(−0.10, −0.02)	0.007
Asian	16	0.173	0.06(−0.02, 0.13)	0.156
Other	8	0.562	−0.03(−0.06, 0.01)	0.536
Healthy	35	0.332	−0.03(−0.07, 0.011)	0.160
CVD	8	0.632	−0.02(−0.13, 0.09)	0.729
Type 2 diabetes	4	0.656	0.01(−0.14, 0.15)	0.915

CVD: cardiovascular disease, TG: triglyceride, TC: total cholesterol, LDL-C: low-density lipoprotein cholesterol, HDL-C: high-density lipoprotein cholesterol.

### Associations of the *APOC3* rs5128 polymorphism with APOC3 and lipid levels

**Figure 1:**
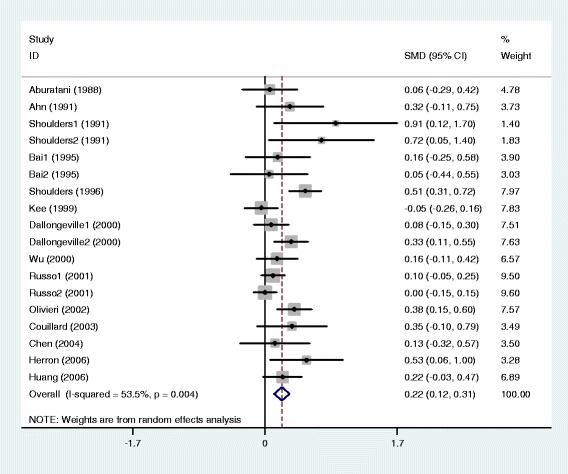
Forest plot of the *APOC3* rs5128 polymorphism and the plasma levels of APOC3 association. Result from the analysis on all 18 comparisons.

**Figure 2:**
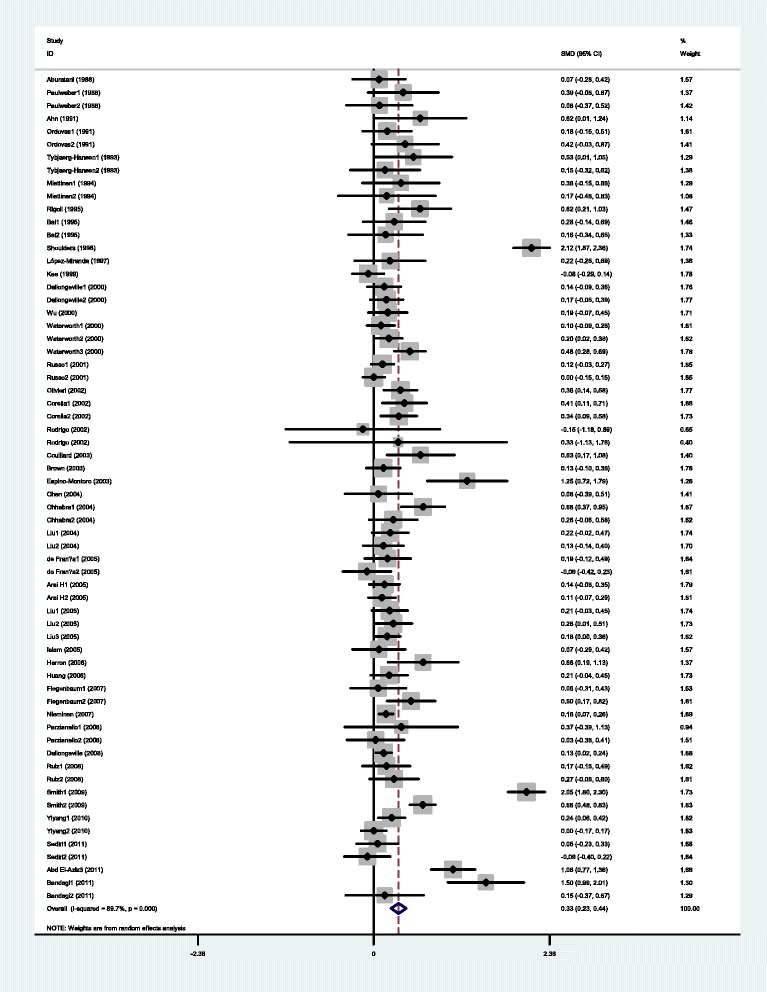
Forest plot of the *APOC3* rs5128 polymorphism and plasma levels of triglycerides association. Result from the analysis on all 64 comparisons.

**Figure 3:**
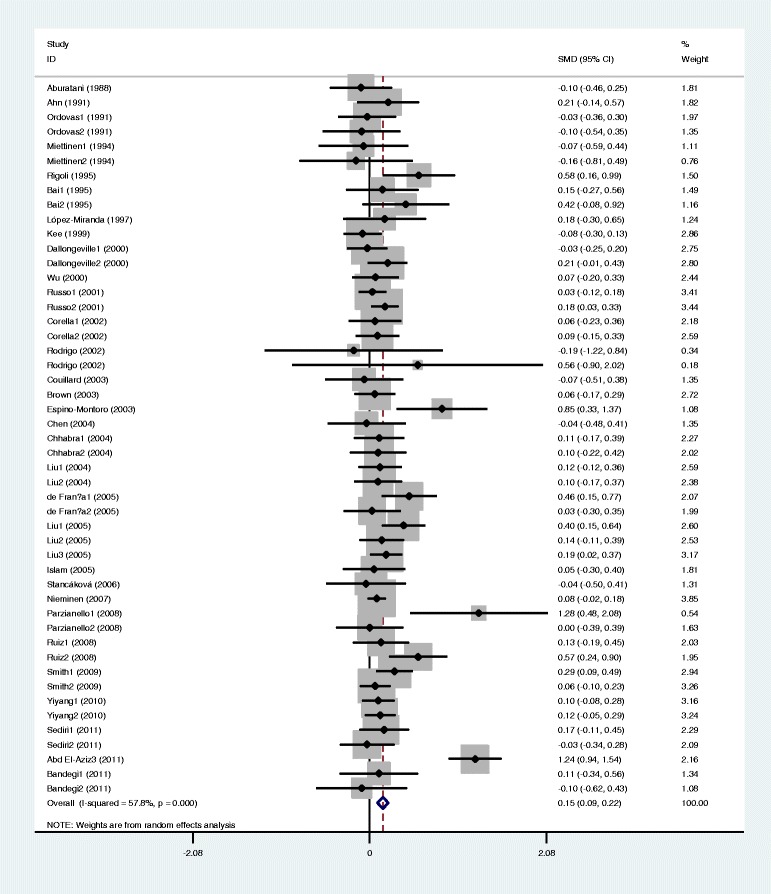
Forest plot of the *APOC3* rs5128 polymorphism and the plasma levels of total cholesterol association. Result from the analysis on all 49 comparisons.

**Figure 4:**
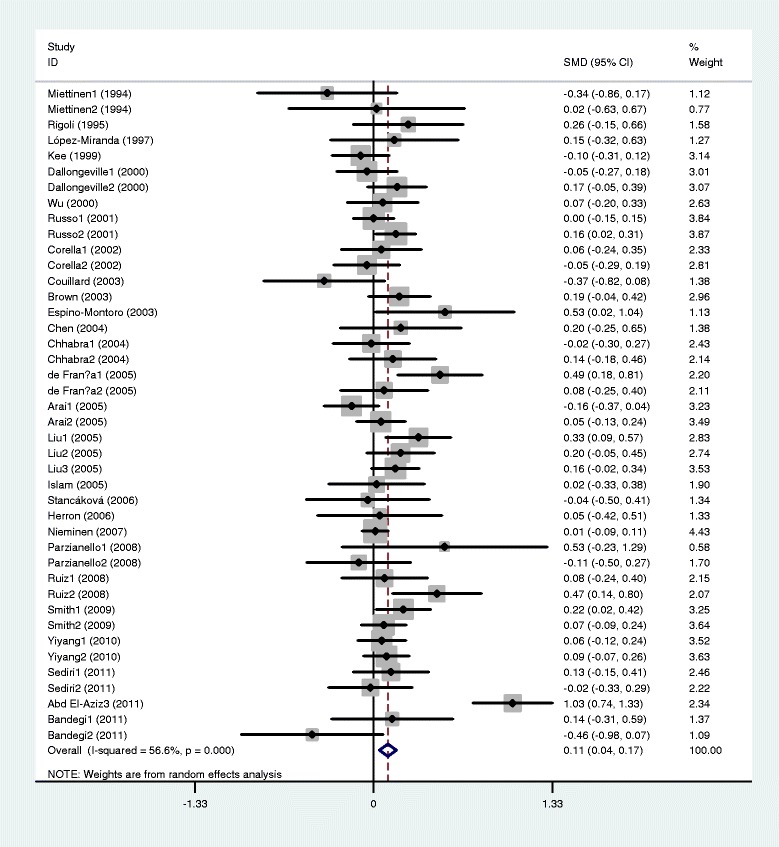
Forest plot of the *APOC3* rs5128 polymorphism and plasma levels of LDL-C association. Result from the analysis on all 42 comparisons.

**Figure 5:**
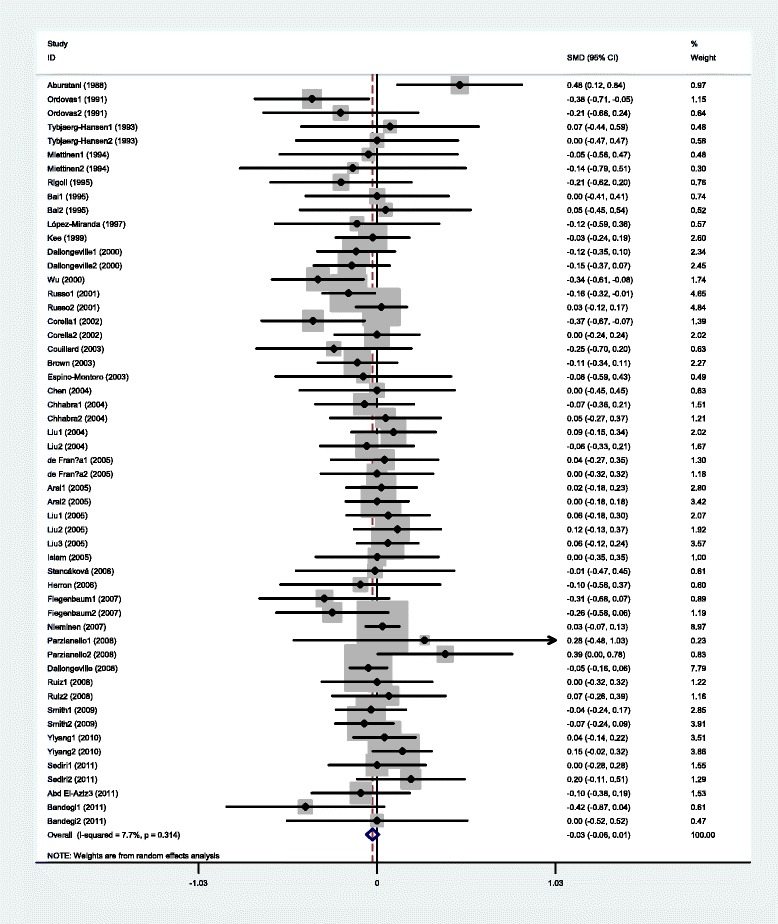
Forest plot of the *APOC3* rs5128 polymorphism and plasma levels of HDL-C association. Result from the analysis on all 54 comparisons.

The outcomes of the analyses on all comparisons showed that the G carriers had higher levels of APOC3 (SMD: 0.22, 95% CI: 0.12-0.31, *P* < 0.00001), TG (SMD: 0.33, 95% CI: 0.23-0.44, *P* < 0.00001), TC (SMD: 0.15, 95% CI: 0.09-0.22, *P* < 0.00001), and LDL-C (SMD: 0.11, 95% CI: 0.04-0.17, *P* = 0.001) than the non-carriers (Table 2, Figures 1, 2, 3, 4). No statistically significant difference in the levels of HDL-C (SMD: −0.03, 95% CI: −0.06-0.01, *P* = 0.156) was detected between the G carriers and the non-carriers (Table 2, Figure 5). In the available studies in HWE, the associations between the *APOC3* rs5128 polymorphism and higher levels of APOC3 (SMD: 0.20, 95% CI: 0.09-0.32, *P* = 0.001), TG (SMD: 0.31, 95% CI: 0.20-0.42, *P* < 0.00001), TC (SMD: 0.12, 95% CI: 0.07-0.18, *P* < 0.00001) and LDL-C (SMD: 0.07, 95% CI: 0.01-0.13, *P* = 0.021) were also significant (Table 2).

Then the subgroup analyses stratified by the characteristics of the subjects were performed. The associations between the *APOC3* rs5128 polymorphism and higher levels of APOC3 and TG were found to be significant across all subpopulations except that in male subjects for APOC3 (SMD: 0.11, 95% CI: −0.05-0.27, *P* = 0.170) and children for TG (SMD: 0.53, 95% CI: −0.37-1.44, *P* = 0.249) (Table 2). The associations between the *APOC3* rs5128 polymorphism and higher TC levels were significant across all subpopulations except that in males (SMD: 0.06, 95% CI: −0.01-0.14, *P* = 0.105) and other ethnic origins (SMD: 0.25, 95% CI: −0.03-0.53, *P* = 0.081). The significant associations between the *APOC3* rs5128 polymorphism and higher LDL-C levels were detected in females (SMD: 0.13, 95% CI: 0.02-0.24, *P* = 0.021), children (SMD: 0.28, 95% CI: 0.05-0.51, *P* = 0.016), Asians (SMD: 0.09, 95% CI: 0.01-0.16, *P* = 0.021), and marginally in Caucasians (SMD: 0.06, 95% CI: 0.00-0.13, *P* = 0.051). The associations between the *APOC3* rs5128 polymorphism and higher levels of LDL-C were also significant in healthy subjects (SMD: 0.06, 95% CI: 0.01-0.11, *P* = 0.012) and diabetic patients (SMD: 0.22, 95% CI: 0.08-0.36, *P* = 0.002) (Table 2). The associations between the *APOC3* rs5128 polymorphism and lower levels of HDL-C were not significant across all subgroups except that in Caucasians (SMD: −0.06, 95% CI: −0.10 to −0.02, *P* = 0.007).

### Heterogeneity analysis and publication bias

There was significant heterogeneity among the total comparisons for APOC3, TG, TC, and LDL-C. Seven comparisons (shoulders, 1996, Russo1, 2001, Dallongeville1, 2000, Aburatani, 1988, Olivieri, 2002, Dallongeville2, 2000, Herron, 2006), 12 comparisons (shoulders, 1996, Smith1, 2009, Smith2, 2009, Abd El-Aziz3, 2011, Parzianello2, 2008, Sediri1, 2011, Dallongeville, 2008, Waterworth1, 2000, Bandegi1, 2011, Espino-Montoro, 2003, Chhabra1, 2004, Waterworth3, 2000), 3 comparisons (Abd El-Aziz3, 2011, Russo1, 2001, Liu1, 2005) and 4 comparisons (Nieminen, 2007, Abd El-Aziz3, 2011, de França1, 2005, Liu1, 2005) were respectively identified as the main contributors of the heterogeneity for APOC3, TG, TC, and LDL-C by using Galbraith plot. The heterogeneity was effectively removed or decreased after exclusion of these outlier studies, but the SMD values andtheir 95% CI did not change significantly (APOC3: SMD: 0.14, 95% CI: 0.03-0.26, *P* = 0.018, *P*_heterogeneity_ = 0.158; TG: SMD: 0.17, 95% CI: 0.14-0.21, *P* < 0.00001, *P*_heterogeneity_ = 0.311; TC: SMD: 0.12, 95% CI: 0.08-0.17, *P* < 0.00001, *P*_heterogeneity_ = 0.152; LDL-C: SMD: 0.07, 95% CI: 0.03-0.12, *P* = 0.002, *P*_heterogeneity_ = 0.270). No significant heterogeneity was found among the total comparisons and the subgroup analyses for HDL-C.

The Egger’s test revealed that no publication bias was present in the analyses for APOC3 (*t*: 1.94, 95% CI: −0.14-3.13, *P* = 0.071), TG (*t*: 1.57, 95% CI: −0.37-3.09, *P* = 0.121), TC (*t*: 1.13, 95% CI: −0.44-1.56, *P* = 0.264), LDL-C (*t*: 0.85, 95% CI: −0.66-1.61, *P* = 0.402), and HDL-C (*t*: −0.85, 95% CI: −0.92-0.38, *P* = 0.402). Figures A-E of the Additional file 2: Figure S1 are the Egger’s plots for APOC3, TG, TC, LDL-C and HDL-C, respectively.

## Discussion

A genome-wide association study has suggested that the polymorphisms in or near the *APOC3* gene are among the strongest genetic determinants of plasma lipid concentrations [55]. A large body of literature has investigated the associations of the *APOC3* rs5128 polymorphism with plasma APOC3 and/or lipid levels [11-52]. Associations of this polymorphism with increased levels of APOC3 [14,20,27,41,42], TG [18,20,23,25,28,30,32,33,38,41,42,44,46,51], TC [18,32,51] and LDL-C [30,51], and/or decreased levels of HDL-C [24] have been reported in some, but not all studies. The lack of consistency across these studies reflects some existed limitations such as small sample size, ethnic differences and research methodology. In the present meta-analysis, the associations of the *APOC3* rs5128 polymorphism with plasma APOC3 and lipid levels were investigated to examine these discrepancies.

The frequencies of the GG homozygote were very low across the populations. Most of the included studies only provided data for CC genotype and G allele carriers (GC + GG), and did not provide data separately for heterozygote CG and homozygote GG. Therefore, a dominant model (CC vs. CG + GG) was employed for this meta-analysis to ensure adequate statistical power. The results suggested that the *APOC3* rs5128 polymorphism was significantly associated with fasting plasma levels of APOC3, TG, TC, and LDL-C under the dominant model. The carriers of G allele had higher levels of APOC3, TG, TC, and LDL-C than the non-carriers. A recent meta-analysis [10] demonstrated that the rs5128 polymorphism is associated with CVD risk. CVD is recognized as a multifactorial disease, and dyslipidemia accounts for at least 50% of the population-attributable risk [2]. Taken our results together, it is possible that the association between the rs5128 polymorphism and CVD is mediated by the dyslipidemia caused by the G allele of the rs5128 polymorphism.

Subgroup analyses by age, gender, ethnicity and health condition were performed since they might be important variables in determining associative risk with dyslipidemia. For example, the present analyses indicated that age might modulate the association between the rs5128 polymorphism and TG levels since the significant association especially exists in males and females, but not in children (Table 2). In addition, the significant effects of the rs5128 polymorphism on TC and LDL-C exist in females and children, but not in males; the significant effect of the polymorphism on LDL-C exists in healthy subjects and diabetic patients, but not in CVD patients. More studies should be conducted to further examine the association of this polymorphism with LDL-C in CVD patients. The associations of the rs5128 polymorphism with plasma levels of APOC3, TG, TC and LDL-C were very robust, which did not vary greatly when the analyses were performed only with the available studies in HWE. However, the significant association of the *APOC3* rs5128 polymorphism with plasma HDL-C levels was not detected in this meta-analysis.

Significant heterogeneity was found across the analyses for APOC3, TG, TC, and LDL-C. The main sources of heterogeneity were from ethnic origin, study design, gender and health condition of the subjects, etc. Subgroup analyses stratified by the characteristics of the subjects were performed to explore the potential source of the observed heterogeneity, and significant heterogeneity was still observed in some subgroups. Galbraith plot was employed to further evaluate the sources of heterogeneity. Outlier studies were identified by using the plot, and the heterogeneity was effectively removed or decreased after exclusion of these outlier studies. No significant changes of the SMD value were found after excluding the outlier studies.

The associations of the *APOC3* rs5128 polymorphism with plasma APOC3, TG, TC and LDL-C were not likely to be type I errors (false-positive results). Firstly, the results from this meta-analysis were based on the random effects model. Comparing with fixed effects model, the random effects model is a more conservative method and less likely to produce false-positive results. Secondly, 42 studies with 23846 subjects were included in this meta-analysis. Among the subjects, 26% of them were the carriers of the rs5128 G allele. Since the incidence of the G allele carriers was sufficiently high, type I error may have been prevented.

The possible mechanism under which the rs5128 polymorphism modulates plasma APOC3 has not been clarified yet. One explanation could be that the G allele enhances the transcriptional activity of *APOC3* and leads to a higher plasma APOC3 level since the rs5128 polymorphism is located in the 3'UTR of exon 4 of this gene. In the present analyses, a significant higher level of APOC3 was found in G carriers comparing with the CC genotype subjects. Previous studies have shown that APOC3 can increase plasma TG levels. Three mechanisms are involved in the elevation of TG levels by APOC3. Firstly, APOC3 promotes the assembly and secretion of VLDL in liver [56-58]. Overexpression of *APOC3* in McA-RH7777 cells by recombinant adenovirus expression vector resulted in significantly increased VLDL assembly and secretion with a dose-dependent effect [56]. APOC3 gene knockout mice showed no increase in VLDL secretion after two weeks of high-fat diet [57]. Functional analysis found that two regions on the APOC3 polypeptide chain are closely related to VLDL synthesis and secretion [57,58]. Secondly, APOC3 inhibits LPL, which is located on the inner side of capillaries and is the main enzyme to hydrolyze TG-rich particles [59]. Thirdly, APOC3 inhibits hepatic lipase. Hepatic lipase is located on the endothelial side of liver sinusoids, and its main function is to remove the remnants of chylomicron and VLDL.

The present meta-analysis also suggested the significant associations of the rs5128 polymorphism with higher levels of LDL-C and TC. There is interrelationship between TG metabolism and cholesterol metabolism in the body, so the elevation of VLDL-TG by APOC3 might have disturbed the cholesterol metabolism and increased plasma LDL-C and TC levels. For example, LDL particles are formed in the bloodstream as VLDL particles lose TG through the action of LPL. Hence, the increase of VLDL Levels can cause the elevation of LDL levels, and accordingly the LDL-C levels. Since approximately 70% of plasma cholesterol molecules are resided in LDL particles, the elevation of LDL-C can lead to higher levels of TC. In addition, it is likely that the rs5128 polymorphism is in linkage disequilibrium with other causative mutations involved in the metabolism of LDL. *APOC3* is a member of the *APOA1/C3/A4/A5* gene cluster which is the key component in modulating lipid metabolism [60]. Several polymorphism sites in the *APOA1/C3/A4/A5* gene cluster have been found to significantly affect plasma LDL-C levels [61].

In the current study, we did not find significant association between the *APOC3* rs5128 polymorphism and lower levels of HDL-C by meta-analysis. One reason could be that HDL is metabolized independently of APOC3 [4,5]. Unlike VLDL and LDL, nascent HDL particles are mainly formed in liver, matured in bloodstream and go back to liver with full load of cholesteryl ester molecules. In this process, HDL is mainly regulated by the proteins such as apolipoprotein A1 (APOA1), ATP-binding cassette transporter A1 (ABCA1) and cholesteryl ester transfer protein (CETP) [62]. Therefore, the *APOC3* rs5128 polymorphism was found to be associated with the plasma levels of APOC3, TG, LDL-C and TC, but not HDL-C.

The limitations of the present meta-analysis should be noted. Firstly, dyslipidemia is involved in a number of genes as well as some environmental factors. However, the interactions of the rs5128 polymorphism with other polymorphic loci or environmental factors on plasma APOC3 and lipid levels have not been investigated in this analysis due to the lack of the original data of the included studies. In other words, the more precise results could have been gained if more detailed individual data were available or the stratification analyses based on the environmental factors such as diet, exercise, and smoking status were performed. Secondly, a relatively small number of subjects were included for the association analysis between the rs5128 polymorphism and plasma APOC3 levels due to the limited available studies, which may reduce the statistic power and even cause the type II errors (false-negative results). More studies with larger sample size are required to further investigate the association. Thirdly, this meta-analysis only included the studies published in English because it was very difficult to get the full papers published in various languages.

## Conclusions

In conclusion, the significant associations between the *APOC3* rs5128 polymorphism and higher levels of APOC3, TG, TC, and LDL-C were found in the present meta-analysis.

## Additional files

Additional file 1: Table S1. Presenting the plasma levels of APOC3 and lipid variables by genotypes of individual studies included in the meta-analysis. Additional file 2: Figure S1. Egger’s plots detecting potential publication bias.
