# Supplementary material for: Associations of the APOC3 rs5128 polymorphism with plasma APOC3 and lipid levels: a meta-analysis
Source: Lipids Health Dis. 2015 Apr 18;14:32. doi: 10.1186/s12944-015-0027-0 (PMC4457007; doi:10.1186/s12944-015-0027-0)
Supplement: Additional file 1: Table S1. — Presenting the plasma levels of APOC3 and lipid variables by genotypes of individual studies included in the meta-analysis. [file 12944_2015_27_MOESM1_ESM.doc]

Table S1. Plasma APOC3 and lipid levels by rs5128 genotype of individual studies included in the meta-analysis

| First author, reference | Genotype | |  | APOC3, mg/dL | |  | TG, mmol/L | |  | TC, mmol/L | |  | LDL-C, mmol/L | |  | HDL-C, mmol/L | |
| --- | --- | --- | --- | --- | --- | --- | --- | --- | --- | --- | --- | --- | --- | --- | --- | --- | --- |
|  | CC | CG+GG |  | CC | CG+GG |  | CC | CG+GG |  | CC | CG+GG |  | CC | CG+GG |  | CC | CG+GG |
| Aburatani [11] | 51 | 77 |  | 11.20±8.20 | 11.89±12.63 |  | 2.01±2.07 | 2.19±2.80 |  | 5.42 ± 1.60 | 5.26 ± 1.52 |  | - | - |  | 1.16 ± 0.35 | 1.37 ± 0.49 |
| Paulweber1 [12] | 84 | 22 |  | - | - |  | 1.58 ± 0.78 | 1.88 ± 0.68 |  | - | - |  | - | - |  | - | - |
| Paulweber2 [12] | 93 | 25 |  | - | - |  | 1.40 ± 0.76 | 1.46 ± 0.92 |  | - | - |  | - | - |  | - | - |
| Ahn [13] | 50 | 42 |  | 6.70±3.89 | 8.05±4.53 |  | 1.48 ± 0.76 | 1.96 ± 0.78 |  | 4.06 ± 1.06 | 4.30 ± 1.15 |  | - | - |  | - | - |
| Shoulders1 [14] | 71 | 7 |  | 8.0 ± 2.0 | 9.8 ± 1.8 |  | - | - |  | - | - |  | - | - |  | - | - |
| Shoulders2 [14] | 66 | 10 |  | 8.1 ± 2.5 | 9.9 ± 2.4 |  | - | - |  | - | - |  | - | - |  | - | - |
| Ordovas1 [15] | 153 | 47 |  | - | - |  | 2.08 ± 1.21 | 2.29 ± 1.04 |  | 5.30 ± 1.19 | 5.26 ± 1.52 |  | - | - |  | 0.88 ± 0.21 | 0.80 ± 0.22 |
| Ordovas2 [15] | 122 | 23 |  | - | - |  | 1.52 ± 1.19 | 2.10 ± 2.13 |  | 5.22 ± 1.06 | 5.12 ± 0.98 |  | - | - |  | 1.29 ± 0.49 | 1.19 ± 0.39 |
| Tybjaerg-Hansen1 [16] | 103 | 17 |  | - | - |  | 1.82 ± 1.06 | 2.39±1.14 |  | - | - |  | - | - |  | 1.18 ± 0.27 | 1.20 ± 0.25 |
| Tybjaerg-Hansen2 [16] | 66 | 24 |  | - | - |  | 1.49 ± 0.66 | 1.61 ± 1.08 |  | - | - |  | - | - |  | 1.26 ± 0.34 | 1.26 ± 0.32 |
| Miettinen1 [17] | 62 | 19 |  | - | - |  | 2.01 ± 1.30 | 2.55 ± 1.99 |  | 6.12 ± 1.35 | 6.02 ± 1.37 |  | 3.82 ± 1.09 | 3.45 ± 1.02 |  | 1.27 ± 0.42 | 1.25 ± 0.37 |
| Miettinen2 [17] | 37 | 12 |  | - | - |  | 1.58 ± 1.09 | 1.81 ± 1.90 |  | 5.70 ± 1.08 | 5.53 ± 0.96 |  | 3.41 ± 0.79 | 3.43 ± 1.06 |  | 1.34 ± 0.37 | 1.29 ± 0.31 |
| Rigoli [18] | 93 | 31 |  | - | - |  | 1.85±0.82 | 2.48±1.46 |  | 5.29±0.92 | 5.86±1.18 |  | 3.44±1.06 | 3.72±1.18 |  | 1.09±0.32 | 1.02±0.38 |
| Bai1 [19] | 43 | 47 |  | 8.4±3.7 | 8.99±3.50 |  | 1.29 ± 0.67 | 1.47±0.64 |  | 4.76 ± 1.06 | 4.91±0.97 |  | - | - |  | 0.96 ± 0.28 | 0.96±0.26 |
| Bai2 [19] | 27 | 38 |  | 10.8±7.5 | 11.12±4.98 |  | 1.26 ± 0.62 | 1.42±1.22 |  | 4.91 ± 1.16 | 5.35±0.95 |  | - | - |  | 1.22 ± 0.36 | 1.24±0.45 |
| Shoulders [20] | 382 | 121 |  | 5.54 ± 1.41 | 6.26±1.37 |  | 0.64 ± 0.02 | 0.75±0.1 |  | - | - |  | - | - |  | - | - |
| López-Miranda [21] | 67 | 23 |  | - | - |  | 0.97 ± 0.47 | 1.08 ± 0.61 |  | 4.19 ± 0.72 | 4.32 ± 0.80 |  | 2.53 ± 0.72 | 2.64 ± 0.70 |  | 1.22 ± 0.26 | 1.19 ± 0.26 |
| Kee [22] | 584 | 100 |  | 3±1.5 | 2.93±1.11 |  | 1.81±1.54 | 1.70 ± 0.79 |  | 6.00±1.22 | 5.90 ± 1.01 |  | 3.98±1.06 | 3.88 ± 0.91 |  | 1.34±0.39 | 1.33 ± 0.36 |
| Dallongeville1 [23] | 406 | 94 |  | 4.8±5.2 | 5.2±4.5 |  | 1.60±1.39 | 1.80±1.63 |  | 5.86±1.05 | 5.83±0.98 |  | 3.85±1.02 | 3.80±1.03 |  | 1.35±0.40 | 1.30±0.47 |
| Dallongeville2 [23] | 407 | 100 |  | 4.0±2.0 | 4.8±3.6 |  | 1.19±1.81 | 1.49±1.57 |  | 5.81±1.04 | 6.03±1.11 |  | 3.62±0.98 | 3.79±0.99 |  | 1.69±0.49 | 1.62±0.42 |
| Wu [24] | 125 | 101 |  | 9±3.35 | 9.66±5.16 |  | 1.11±1.01 | 1.29±0.88 |  | 4.86±0.87 | 4.92±0.93 |  | 2.92±0.87 | 2.98±0.92 |  | 1.45±0.58 | 1.27±0.45 |
| Waterworth1 [25] | 670 | 133 |  | - | - |  | 1.52±0.72 | 1.59±0.81 |  | - | - |  | - | - |  | - | - |
| Waterworth2 [25] | 844 | 137 |  | - | - |  | 1.62±0.83 | 1.79±0.87 |  | - | - |  | - | - |  | - | - |
| Waterworth3 [25] | 609 | 106 |  | - | - |  | 1.68±0.81 | 2.10±1.14 |  | - | - |  | - | - |  | - | - |
| Russo1 [26] | 1041 | 201 |  | 16.2 ± 4.8 | 16.7 ± 5.2 |  | 1.51 ± 1.07 | 1.64 ± 1.34 |  | 5.25 ± 0.96 | 5.28 ± 0.96 |  | 3.44 ± 0.88 | 3.44 ± 0.85 |  | 1.14 ± 0.31 | 1.09 ± 0.27 |
| Russo2 [26] | 1054 | 212 |  | 16.5 ± 4.3 | 16.5 ± 3.9 |  | 1.22 ± 0.96 | 1.22 ± 0.73 |  | 5.25 ± 1.01 | 5.43 ± 1.01 |  | 3.26 ± 0.91 | 3.41 ± 0.93 |  | 1.44 ± 0.39 | 1.45 ± 0.40 |
| Olivieri [27] | 438 | 96 |  | 11.3±3.8 | 12.8±4.8 |  | 1.75 ± 0.92 | 2.10 ± 1.22 |  | - | - |  | - | - |  | - | - |
| Corella1 [28] | 309 | 51 |  | - | - |  | 1.32 ± 0.71 | 1.62 ± 0.85 |  | 5.22 ± 0.99 | 5.28 ± 0.86 |  | 3.48 ± 0.88 | 3.53 ± 0.75 |  | 1.13 ± 0.24 | 1.04 ± 0.26 |
| Corella2 [28] | 471 | 75 |  | - | - |  | 0.87 ± 0.45 | 1.03 ± 0.62 |  | 5.00 ± 0.89 | 5.08 ± 0.90 |  | 3.17 ± 0.79 | 3.13 ± 0.77 |  | 1.43 ± 0.26 | 1.43 ± 0.32 |
| Rodrigo1 [29] | 37 | 4 |  | - | - |  | 1.47 ± 0.70 | 1.37 ± 0.37 |  | 5.12 ± 1.53 | 4.84 ± 0.49 |  | - | - |  | - | - |
| Rodrigo2 [29] | 21 | 2 |  | - | - |  | 1.25 ± 0.80 | 1.52 ± 1.22 |  | 5.20 ± 1.99 | 6.31 ± 1.73 |  | - | - |  | - | - |
| Couillard [30] | 98 | 24 |  | 14.4 ± 3.6 | 15.6 ± 2.9 |  | 2.34 ± 0.95 | 3.03 ± 1.58 |  | 5.43 ± 0.77 | 5.38 ± 0.66 |  | 3.71 ± 0.72 | 3.45 ± 0.66 |  | 0.88 ± 0.16 | 0.84 ± 0.16 |
| Brown [31] | 205 | 115 |  | - | - |  | 1.35 ± 0.86 | 1.46 ± 0.86 |  | 4.57 ± 0.86 | 4.62 ± 0.86 |  | 2.69 ± 0.72 | 2.83 ± 0.75 |  | 1.08 ± 0.29 | 1.05 ± 0.21 |
| Espino-Montoro [32] | 86 | 18 |  | - | - |  | 1.5±1.1 | 2.9±1.2 |  | 5.52±1.25 | 6.6+1.4 |  | 3.4±1.1 | 4.0+1 3 |  | 1.23+0.4 | 1.2±0.2 |
| Chen [33] | 34 | 44 |  | 11.64 ± 4.21 | 12.29 ± 5.76 |  | 1.37 ± 0.56 | 1.42±0.95 |  | 4.81 ± 1.16 | 4.77 ± 1.05 |  | 2.77 ± 0.80 | 2.95 ± 0.96 |  | 1.17 ± 0.33 | 1.17 ± 0.41 |
| Chhabra1 [34] | 85 | 108 |  | - | - |  | 1.59±0.66 | 2.08±0.80 |  | 4.93±1.38 | 5.08±1.36 |  | 3.16 ± 1.15 | 3.14 ± 1.13 |  | 1.00 ± 0.30 | 0.98 ± 0.25 |
| Chhabra2 [34] | 71 | 80 |  | - | - |  | 1.46±0.84 | 1.68±0.84 |  | 4.98±1.01 | 5.08±1.03 |  | 2.86 ± 0.93 | 2.99 ± 0.90 |  | 1.14 ± 0.22 | 1.15 ± 0.19 |
| Liu1 [35] | 295 | 83 |  | - | - |  | 2.21 ± 1.58 | 2.56±1.57 |  | 5.82 ± 0.96 | 5.94±1.12 |  | - | - |  | 1.16 ± 0.31 | 1.19 ± 0.34 |
| Liu2 [35] | 297 | 64 |  | - | - |  | 1.69 ± 1.26 | 1.86±1.57 |  | 5.48 ± 0.93 | 5.57±0.90 |  | - | - |  | 1.29 ± 0.34 | 1.27 ± 0.33 |
| de França1 [36] | 168 | 53 |  | - | - |  | 0.89±0.37 | 0.96±0.40 |  | 3.92±0.60 | 4.21±0.71 |  | 2.37±0.53 | 2.64±0.60 |  | 1.13±0.22 | 1.14±0.27 |
| de França2 [36] | 144 | 49 |  | - | - |  | 1.00±0.44 | 0.96±0.42 |  | 4.13±0.73 | 4.15±0.70 |  | 2.50±0.63 | 2.55±0.65 |  | 1.16±0.25 | 1.16±0.25 |
| Arai1 [37] | 165 | 212 |  | - | - |  | 1.50 ± 0.94 | 1.64 ± 1.01 |  | - | - |  | 3.16 ± 0.92 | 3.02±0.80 |  | 1.37 ± 0.40 | 1.38±0.40 |
| Arai2 [37] | 207 | 268 |  | - | - |  | 1.05 ± 0.78 | 1.16 ± 1.14 |  | - | - |  | 2.90 ± 0.92 | 2.95±0.92 |  | 1.65 ± 0.40 | 1.65±0.37 |
| Liu1 [38] | 130 | 137 |  | - | - |  | 1.77 ± 0.91 | 2.10 ± 1.96 |  | 4.21 ± 1.00 | 4.63±1.11 |  | 2.13 ± 0.73 | 2.39±0.83 |  | 1.13 ± 0.31 | 1.15±0.32 |
| Liu2 [38] | 118 | 128 |  | - | - |  | 1.70 ± 1.38 | 2.11 ± 1.73 |  | 4.56 ± 1.35 | 4.74±1.23 |  | 2.68 ± 0.92 | 2.87±0.98 |  | 1.21 ± 0.40 | 1.26±0.44 |
| Liu3 [38] | 243 | 248 |  | - | - |  | 1.78 ± 1.05 | 1.98 ± 1.16 |  | 5.02 ± 0.91 | 5.19±0.85 |  | 3.02 ± 0.86 | 3.18±1.10 |  | 1.59 ± 0.67 | 1.64±0.93 |
| Islam [39] | 177 | 37 |  | - | - |  | 1.15± 1.62 | 1.26 ± 1.60 |  | 5.17 ± 0.98 | 5.22 ± 1.11 |  | 3.33 ± 0.88 | 3.35 ± 0.98 |  | 1.27± 0.33 | 1.27 ± 0.33 |
| Stancáková [40] | 92 | 23 |  | - | - |  | - | - |  | 5.56 ± 12.09 | 5.07 ± 5.90 |  | 3.40 ± 10.26 | 2.99 ± 5.18 |  | 1.02 ± 2.78 | 0.99 ± 1.49 |
| Herron [41] | 65 | 25 |  | 12.3 ± 3.5 | 14.49 ± 5.43 |  | 0.99 ± 0.50 | 1.40 ± 0.86 |  | - | - |  | 2.41 ± 0.68 | 2.44 ± 0.60 |  | 1.43 ± 0.32 | 1.40 ± 0.29 |
| [Huang](http://www.ncbi.nlm.nih.gov/pubmed?term=Huang MC%5BAuthor%5D&cauthor=true&cauthor_uid=16864937) [42] | 127 | 122 |  | 8.2±3.6 | 9.15 ± 4.82 |  | 1.56±1.10 | 1.87 ± 1.84 |  | - | - |  | - | - |  | - | - |
| Fiegenbaum1 [43] | 145 | 34 |  | - | - |  | 1.55±0.84 | 1.60±0.87 |  | - | - |  | - | - |  | 1.09±0.26 | 1.01±0.27 |
| Fiegenbaum2 [43] | 210 | 45 |  | - | - |  | 1.32±0.72 | 1.70±0.93 |  | - | - |  | - | - |  | 1.23±0.28 | 1.16±0.22 |
| Nieminen [44] | 1674 | 543 |  | - | - |  | 1.30±0.79 | 1.44±1.02 |  | 5.14 ± 0.96 | 5.22 ± 1.07 |  | 3.27 ± 0.84 | 3.28 ± 0.91 |  | 1.29 ± 0.31 | 1.30±0.33 |
| [Parzianello1](http://www.ncbi.nlm.nih.gov/pubmed?term=Parzianello L%5BAuthor%5D&cauthor=true&cauthor_uid=18560672) [45] | 8 | 43 |  | - | - |  | 3.04 ± 1.04 | 3.40±0.97 |  | 4.73 ± 0.67 | 5.61 ± 0.69 |  | 2.40 ± 0.62 | 2.99 ± 1.18 |  | 1.11 ± 0.26 | 1.17±0.21 |
| Parzianello2 [45] | 43 | 65 |  | - | - |  | 1.61 ± 0.42 | 1.62±0.37 |  | 4.99 ± 0.80 | 4.99 ± 0.91 |  | 3.05 ± 0.72 | 2.96 ± 0.87 |  | 1.19 ± 0.28 | 1.30±0.28 |
| [Dallongeville](http://www.ncbi.nlm.nih.gov/pubmed?term=Dallongeville J%5BAuthor%5D&cauthor=true&cauthor_uid=18789138) [46] | 1799 | 407 |  | - | - |  | 1.27 ± 1.4 | 1.45±1.20 |  | - | - |  | - |  |  | 1.49 ± 0.4 | 1.47±0.40 |
| Ruiz1 [47] | 214 | 46 |  | - | - |  | 0.80 ± 0.36 | 0.86 ± 0.35 |  | 4.04 ± 0.68 | 4.13 ± 0.70 |  | 2.36 ± 0.61 | 2.41 ± 0.65 |  | 1.32 ± 0.24 | 1.32 ± 0.29 |
| Ruiz2 [47] | 198 | 44 |  | - | - |  | 0.72 ± 0.29 | 0.80 ± 0.31 |  | 4.32 ± 0.65 | 4.70 ± 0.75 |  | 2.47 ± 0.58 | 2.75 ± 0.66 |  | 1.53 ± 0.29 | 1.55 ± 0.31 |
| [Smith1](http://www.ncbi.nlm.nih.gov/pubmed?term=Smith CE%5BAuthor%5D&cauthor=true&cauthor_uid=19424489) [48] | 215 | 169 |  | - | - |  | 1.62 ± 0.15 | 1.91 ± 0.13 |  | 4.29 ± 1.32 | 4.67 ± 1.30 |  | 2.40 ± 1.03 | 2.58 ± 0.39 |  | 1.06 ± 0.29 | 1.05 ± 0.26 |
| Smith2 [48] | 318 | 239 |  | - | - |  | 1.57 ± 0.18 | 1.68 ± 0.15 |  | 4.76 ± 1.25 | 4.84 ± 1.24 |  | 2.78 ± 1.07 | 2.86 ± 1.08 |  | 1.15 ± 0.36 | 1.12 ± 0.46 |
| Yiyang1 [49] | 212 | 278 |  | - | - |  | 0.82 ± 0.48 | 0.96 ± 0.65 |  | 4.44 ± 0.78 | 4.53 ± 0.98 |  | 2.22 ± 0.62 | 2.26 ± 0.67 |  | 2.11 ± 0.44 | 2.13 ± 0.49 |
| Yiyang2 [49] | 256 | 284 |  | - | - |  | 1.05 ± 0.65 | 1.05 ± 0.62 |  | 4.74 ± 0.95 | 4.86 ± 1.00 |  | 2.51 ± 0.75 | 2.58 ± 0.73 |  | 1.97 ± 0.43 | 2.04 ± 0.52 |
| Sediri1 [50] | 260 | 60 |  | - | - |  | 1.88±0.82 | 1.92±0.82 |  | 4.99±1.13 | 5.18±1.08 |  | 3.31±1.03 | 3.44±0.93 |  | 0.85±0.23 | 0.85±0.23 |
| Sediri2 [50] | 312 | 46 |  | - | - |  | 1.54±0.92 | 1.46±0.84 |  | 4.84±0.95 | 4.81±1.03 |  | 2.97±0.82 | 2.95±0.90 |  | 1.13±0.31 | 1.19±0.28 |
| Abd El-Aziz3 [51] | 240 | 60 |  | - | - |  | 2.02±0.67 | 2.76±0.79 |  | 5.37±1.29 | 7.17±1.99 |  | 3.30±1.23 | 4.75±1.96 |  | 1.16±0.21 | 1.14±0.21 |
| Bandegi1 [52] | 39 | 37 |  | - | - |  | 4.28±0.85 | 5.46±0.71 |  | 7.58 ± 1.45 | 7.73±1.35 |  | 3.90 ± 0.97 | 4.04±1.06 |  | 0.94 ± 0.05 | 0.91±0.09 |
| Bandegi2 [52] | 56 | 19 |  | - | - |  | 1.73±0.25 | 1.77±0.33 |  | 3.41 ± 0.99 | 3.31±1.22 |  | 1.84± 0.74 | 1.53±0.44 |  | 1.16 ± 0.15 | 1.16±0.22 |

TG: triglyceride, TC: total cholesterol, LDL-C: low-density lipoprotein cholesterol, HDL-C: high-density lipoprotein cholesterol.
