# Supplementary material for: Associations of the APOC3 rs5128 polymorphism with plasma APOC3 and lipid levels: a meta-analysis
Source: Lipids Health Dis. 2015 Apr 18;14:32. doi: 10.1186/s12944-015-0027-0 (PMC4457007; doi:10.1186/s12944-015-0027-0)
Supplement: Additional file 2: Figure S1. — Egger’s plots detecting potential publication bias. [file 12944_2015_27_MOESM2_ESM.doc]

A B

C D

E

**Egger’s plots for publication bias test.** A: The plot comparing the difference in APOC3; B: The plot comparing the difference in TG; C: The plot comparing the difference in TC; D: The plot comparing the difference in LDL-C; E: The plot comparing the difference in HDL-C.
